# Typical and Atypical Enteroaggregative *Escherichia coli* Are Both Virulent in the *Galleria mellonella* Model

**DOI:** 10.3389/fmicb.2019.01791

**Published:** 2019-08-13

**Authors:** Caroline Gastaldi Guerrieri, Monalessa Fábia Pereira, Anna Clara Milesi Galdino, André Luis Souza dos Santos, Waldir Pereira Elias, Ricardo Pinto Schuenck, Liliana Cruz Spano

**Affiliations:** ^1^Laboratory of Virology and Infectious Gastroenteritis, Department of Pathology, Health Sciences Center, Federal University of Espírito Santo, Vitória, Brazil; ^2^Laboratory of Advanced Studies of Emerging and Resistant Microorganisms, Department of General Microbiology, Institute of Microbiology Paulo de Góes, Federal University of Rio de Janeiro, Rio de Janeiro, Brazil; ^3^Laboratory of Bacteriology, Butantan Institute, São Paulo, Brazil

**Keywords:** enteroaggregative *E. coli*, bacterial pathogenesis, *Galleria mellonella*, infection model, AggR regulon

## Abstract

Enteroaggregative *Escherichia coli* (EAEC) is an emerging pathotype responsible for acute and persistent diarrhea. It can be classified as typical and atypical strains, respectively, based on the presence or absence of the AggR regulon, suggesting a higher virulence for typical EAEC. This study aims to evaluate in the *Galleria mellonella* model if there are differences in the virulence profiles among clinical strains of typical and atypical EAEC, prototype strains EAEC C1096, 042 and its *aggR* mutant. The clinical EAEC strains (*n* = 20) were analyzed for the presence of 22 putative virulence factors of EAEC or extraintestinal *E. coli* by PCR, as well as phenotypic characteristics of virulence (enzymes, siderophore, and biofilm). The survival of the larvae was analyzed after inoculation of 10^4^–10^7^ CFU/larva; the monitoring of bacterial growth *in vivo* and hemocyte quantification was determined after inoculation of the prototype strains (10^5^ CFU/larva) at different periods after infection. The strains of typical and atypical EAEC presented the same virulence profile for the larva, regardless of the amount or type of genes and phenotypic aspects of virulence analyzed. In addition, the EAEC 042 *aggR* mutant strain showed a significant reduction in the mortality of the inoculated larvae compared to the wild-type strain. In conclusion, the results obtained herein demonstrate that the virulence of EAEC seems to be related to the AggR regulon, but not exclusively, and atypical EAEC strains may be as virulent as typical ones *in vivo* in the *G. mellonella* model.

## Introduction

Enteroaggregative *Escherichia coli* (EAEC), one of the six diarrheagenic pathotypes of *E. coli*, has been the most frequent cause of acute and persistent diarrhea in people of all ages in several regions of the world, including Brazil, corresponding to between 21 and 25% of cases (Moreno et al., 2010; Okhuysen and Dupont, 2010; Lozer et al., 2013; Spano et al., 2017). EAEC, a heterogeneous pathogen that remains misunderstood, has a proposed pathogenesis comprising initial adhesion, biofilm formation, induction of an inflammatory response, and release of toxins; however, putative virulence factors are not uniformly present in all isolated strains (Okeke et al., 2000, 2010; Navarro-Garcia et al., 2010; Jensen et al., 2014).

Presence of AggR regulon distinguishes typical from atypical EAEC, since it regulates both chromosomal and plasmid-encoded virulence factors. In this sense, typical EAEC is suggested to be more virulent and more likely to cause diarrhea in humans than atypical EAEC (Sarantuya et al., 2004; Morin et al., 2013). However, atypical EAEC has also been reported as an enteric pathogen of significant outbreaks (Čobeljić et al., 1996; Itoh et al., 1997). In general, the typical EAEC strains are more frequent than the atypical EAEC, but, interestingly, some studies have demonstrated a higher frequency for atypical, varying from between 57.3 and 73.7%, compared to the typical ones, that range from between 26.3 and 42.7% (Huang et al., 2003, 2007; Opintan et al., 2010; Spano et al., 2017). There is still a gap in understanding their virulence aspects and we believe that well-established *in vivo* models may contribute to elucidate a possible difference between these two groups.

For ethical reasons, studies with human volunteers that would best reproduce bacterial virulence is not currently feasible (Nataro et al., 1995), therefore, studies make use of animal models, mainly rodents, as a way to investigate aspects of the EAEC pathogenesis and, in recent years, for ethical and costs reasons, the use of invertebrate models has been implemented for studies of several microorganisms, even for EAEC (Morin et al., 2010; Roche et al., 2010; França et al., 2013; Jønsson et al., 2016; Khalil et al., 2016). In this context, the larvae of *Galleria mellonella* (Lepidoptera: Pyralidae) shows several morphological, physiological and immunological advantages over other invertebrate models (Tsai et al., 2016). Their relatively larger size (between 12 and 20 mm), in comparison to other invertebrate models, makes the larvae easier to handle for injecting the inoculum directly into the larval haemocoel, and removing tissues and hemolymph for subsequent analysis (Cook and Mcarthur, 2013). Besides, the survival of *G. mellonella* at 37°C allows investigating temperature-dependent virulence factors, and its immune system shares a high degree of structural and functional similarities to the innate immune system of vertebrates, being hemocytes analogous to human phagocytes (Ramarao et al., 2012; Tsai et al., 2016). In addition to the hemocyte-mediated cellular immune response, *G. mellonella* also has a humoral immune response consisting of effector molecules, including opsonins (Park et al., 2005; Shaik and Sehnal, 2009), antimicrobial peptides (Lee et al., 2004; Brown et al., 2009), and melanin (Soderhall and Cerenius, 1998). Previously, Jønsson et al. (2016) and Khalil et al. (2016) analyzed only typical EAEC strains in this same model, comparing it with non-pathogenic *E. coli* and validating the model for EAEC pathogenicity studies.

Therefore, we analyzed, in the *G. mellonella* model, if there are differences in virulence profiles among prototype and clinical strains of both typical and atypical EAEC, duly characterized for phenotypic and genotypic aspects, and mutant strain for the *aggR* gene.

## Materials and Methods

### Bacterial Strains

Twenty EAEC strains were analyzed, ten typical and ten atypical, obtained from stool samples of children up to 11 years of age, with (*n* = 4) and without diarrhea (*n* = 16), during a previous epidemiological study conducted from August 2007 to September 2008 in a rural area of Southeastern Brazil (Lozer et al., 2013). EAEC was identified by its aggregative adherence pattern in a HEp-2 cell culture; typical and atypical EAEC strains were classified according to the presence or absence of *aggR*, respectively (Lozer et al., 2013). Bacterial cultures were kept at −20°C in 24% (w/v) sucrose solution.

The prototype strains EAEC 042 (typical), C1096 (atypical), non-pathogenic *E. coli* strain HB101 and an *aggR* isogenic mutant of EAEC 042 (Nataro et al., 1995; Čobeljić et al., 1996; Elias et al., 1999) were all included in the study. EAEC 042 and HB101 were kindly provided by Dr. Isabel Scaletsky (Federal University of São Paulo, Brazil).

### Genotypic and Phenotypic Characterization of EAEC Strains

#### Detection of Virulence Genes by Polymerase Chain Reaction (PCR)

The 20 EAEC strains were screened by PCR for the presence of 17 putative virulence factors described for EAEC as those related to bacterial adhesion, toxin production, iron uptake, and biofilm production, for which the target genes, sequences of primers, amplicon sizes, and control strains are shown in Table 1. Besides that, all EAEC strains, including the prototypes EAEC 042 and C1096, were analyzed for extraintestinal virulence markers to detect some virulence genes used to classify *E. coli* strains as extraintestinal pathogenic *E. coli* (ExPEC), according to Johnson et al. (2003), i.e., *papC*, *sfa*, *afaC*, *iutA*, and *kpsMTII*.

**TABLE 1:** Primers, amplicon sizes, and control strains to virulence genes detection.

**Gene**	**Encoded protein**	**Primer pair (5′–3′)**	**Control strain**	**Amplicon size (pb)**	**References**
*aggA*	AAF/I fimbrial subunit	TTAGTCTTCTATCTAGGG AAATTAATTCCGGCATGG	EAEC 17-2	450	Czeczulin et al., 1999
*aafA*	AAF/II fimbrial subunit	ATGTATTTTTAGAGGTTGAC TATTATATTGTCACAAGCTC	EAEC 042	518	
*agg3A*	AAF/III fimbrial subunit	GTATCATTGCGAGTCTGGTATTCAG GGGCTGTTATAGAGTAACTTCCAG	*E. coli* RN785-1	462	Bernier et al., 2002
*agg4A*	AAF/IV fimbrial subunit	TCCATTATGTCAGGCTGCAA GGCGTTAACGTCTGATTTCC	Personal collection	411	Boisen et al., 2008
*aggR*	EAEC transcriptional activator	CTAATTGTACAATCGATGTA ATGAAGTAATTCTTGAAT	EAEC 042	308	Czeczulin et al., 1999
*aap*	Antiaggregation protein (dispersin)	CTTTTCTGGCATCTTGGGT GTAACAACCCCTTTGGAAGT	EAEC 042	232	
*shf*	Cryptic ORF	ACTTTCTCCCGAGACATTC CTTTAGCGGGAGCATTCAT	EAEC 042	613	
*hlyA*	α-haemolysin	CTCATTGGCCTCACCGAACGG GCTGGCAGCTGTGTCCACGAG	Personal collection	299	Guignot et al., 2000
*irp2*	Yersiniabactin biosynthetic gene	AAGGATTCGCTGTTACCGGAC TCGTCGGGCAGCGTTTCTTCT	*E. coli* RN785-1	264	Czeczulin et al., 1999
*sen*	*Shigella* enterotoxin2	ATGTGCCTGCTATTATTTAT CATAATAATAAGCGGTCAGC	Personal collection	799	Vila et al., 2000
*agn43*	Antigen 43	ACGCACAACCATCAATAAAA CCGCCTCCGATACTGAATGC	EAEC 042	600	Mendez-Arancibia et al., 2008
*sat*	Secreted auto transporter toxin	ACTGGCGGACTCATGCTGT AACCCTGTAAGAAGACTGAGC	*Shigella flexneri* MA245-5	387	Ruiz et al., 2002
*astA*	EAEC heat-stable enterotoxin 1	CCATCAACACAGTATATCCGA GGTCGCGAGTGACGGCTTTGT	EAEC 042	111	Huang et al., 2007^*^
*pet*	Plasmid encoded toxin	GACCATGACCTATACCGACAGC CCGATTTCTCAAACTCAAGACC	EAEC 042	600	
*set1A*	*Shigella* enterotoxin1	TCACGCTACCATCAAAGA TATCCCCCTTTGGTGGTA	EAEC 042	309	
*chuA*	*E. coli* haem utilization gene	ATCTGCTGCGTCATGTTCCT GTAGTGGTCATACCTTTGAGC	*E. coli* EDL933	1700	Okeke et al., 2004^*^
*iucA*	Aerobactin siderophore	AGTCTGCATCTTAACCTTCA CTCGTTATGATCGTTCAGAT	*Shigella flexneri* MA245-5	1100	
*afaC*	Afa/Dr adhesin	CGGCTTTTCTGCTGAACTGGCAGGC CCGTCAGCCCCCACGGCAGACC	C1845	672	Le Bouguenec et al., 2001
*papC*	P fimbriae	GACGGCTGTACTGCAGGGTGTGGCG ATATCCTTTCTGCAGGGATGCAATA	*E. coli* J96	328	Le Bouguenec et al., 1992^*^
*sfa*	S fimbriae	CTCCGGAGAACTGGGTGCATCTTAC CGGAGGAGTAATTACAAACCTGGCA	*E. coli* J96	410	
*iutA*	Aerobactin receptor	GGCTGGACATCATGGGAACTGG CGTCGGGAACGGGTAGAATCG	*Shigella flexneri* SA101	300	Johnson and Stell, 2000^*^
*kpsMTII*	Group 2 capsule synthesis	GCGCATTTGCTGATACTGTTG CATCCAGACGATAAGCATGAGCA	*E. coli* HB101	272	

^*^Multiplex PCR.

Bacterial DNA was extracted by boiling a suspension of 1 colony in 50 μl of distilled water. All the reactions were carried out in a final volume of 25 μl with 5 μl of extracted DNA (10 ng/μl), reaction buffer, 1.5 mm of MgCl_2_, 200 μm of each dNTP, 0.02 U/μl of DNA Taq polymerase^TM^ (Invitrogen) and 0.4 μm of each primer, except for 0.16 μm of *set1A*, *astA*, and *pet* primers used in a multiplex reaction (Table 1), 0.3 μm of *kpsMTII* and 0.6 for *iutA*.

Amplifications were performed in a Veriti^®^ Thermal Cycler (Applied Biosystems^®^) and the amplicons observed after electrophoresis in 1.5% agarose gels in Tris-borate-EDTA (TBE) buffer 0.5X, stained with ethidium bromide.

#### Siderophore and Hydrolytic Enzymes Production Assays

The production of siderophore and hydrolytic enzymes (phospholipase, esterase, protease, and hemolysin) were investigated, starting from 200 μl of bacterial suspension at the concentration of 1.5 × 10^8^ CFU/ml, added to 2 ml of Luria Bertani (LB) (Kasvi) broth and incubated at 37°C for 24 h. Then, 5 μl of the suspension were applied to the center of Petri dishes with chrome azurol S agar (CAS) for siderophore assay or culture media containing the substrate for the hydrolytic enzyme to be tested, followed by incubation at 37°C for 48 h. The culture media compositions were: (i) Phospholipase – glucose 20 g/L, yeast extract 5 g/L, peptone 10 g/L, NaCl 40 g/L CaCl_2_ 0.74 g/L, bacteriological agar 15 g/L, and 2% (w/v) egg yolk; (ii) Protease – tryptone 10 g/L, NaCl 10 g/L, yeast extract 5 g/L, bacterial agar 12 g/L + 1% (w/v) skimmed milk powder; (iii) Esterase – peptone 10 g/L, NaCl 5 g/L, CaCl_2_.2H_2_O 0.1 g/L, bacteriological agar 15 g/L Tween 80 10 ml/L; (iv) Hemolysin – blood agar 5% sheep red blood cell – Merckoplate^®^. Siderophore production was observed by the formation of an orange halo around the colony, and enzymatic activity by the formation of a halo of degradation. All assays were quantified by the ratio between colony and halo diameter (*D*_colony_/*D*_halo_) and classified as a weak, good or excellent producer, as previously described (Price et al., 1982; Searle et al., 2015; Ramos et al., 2016).

#### Biofilm Formation Assay

The biofilm formation assay was performed as described by Sheikh et al. (2001), with some modifications. In summary, a colony of each strain obtained on nutrient agar (Kasvi) was suspended in 2 ml of LB broth and incubated at 37°C for 18 h at 110 rpm. The suspension was then adjusted to a concentration of 1.5 × 10^8^ CFU/ml, and 2 μl of it was added to 200 μl of Dulbecco’s Modified Eagle’s Medium (DMEM) (Cultilab) supplemented with 0.4% (w/v) of glucose in a 96-well polystyrene microplate. The microplates were incubated at 37°C for 24 h for biofilm formation, which was fixed with methanol and stained with crystal violet. Bound crystal violet was solubilized by adding 100 μl of 97% ethanol in ether (v/v) and the biofilm was quantified by absorbance measure at 540 nm (EZ Read 400 microplate reader). Biofilm classification as “forming” and “non-forming” was performed according to Stepanovic et al. (2007).

### *In vivo* Assays in *G. mellonella* Model

#### *G. mellonella* Killing Assay

All EAEC clinical and prototype strains at mid-exponential phase were injected into *G. mellonella* larvae in concentrations ranging from 10^4^ to 10^7^ CFU/larva. The experiments were carried out according to Ramarao et al. (2012). Each concentration was tested on 20 last-instar larvae, each weighing 250–300 mg, and each larva was inoculated with 10 μl of bacterial suspension into the haemocoel using insulin syringes (Becton Dickinson). Infected larvae were placed in Petri dishes, incubated at 37°C, and mortality was monitored every 24 h for up to 96 h after infection. Larvae that did not respond to touch were considered dead. The negative control consisted of larvae inoculated with sterile PBS and non-pathogenic *E. coli* strain HB101. The experiment was performed in triplicate on different days. The strains were classified as highly virulent when less than 40% of the larvae survived at the end of the experiment with 10^5^ CFU/larva, and as moderate or low virulence when between 40 and 60% and more than 60% of the larvae survived, respectively.

#### Monitoring of Bacterial Growth *in vivo*

Bacterial growth monitoring was performed for the prototype strains (EAEC C1096, 042 and its *aggR* mutant) and the non-pathogenic *E. coli* strain HB101, as described by Pereira et al. (2015). The strains were inoculated into haemocoel of *G. mellonella* larvae at the concentration of 10^5^ CFU/larva. The concentration of bacteria into the hemolymph was monitored at 0, 2, 4, 6, and 24 h after infection. At each time, 10 μl of hemolymph were removed by a cut near the pseudo legs after larval surface antisepsis with 70% ethanol, and 10-fold dilutions of each strain were plated on MacConkey agar (Oxoid) and incubated at 37°C for 24 h. The negative control consisted of larvae inoculated with sterile PBS. This procedure was performed for three larvae individually, for each strain at each time, and in a total of three replicates on different days. To calculate the bacterial concentration in CFU, the mean of the tested larvae was considered.

#### Hemocyte Quantification During Infection

Hemocyte quantification was performed according to Pereira et al. (2015). Briefly, 10 μl of hemolymph, obtained from the inoculated larvae as described above, were transferred to microcentrifuge tubes previously treated with Sigmacote (Sigma-Aldrich) containing anticoagulant solution with pH 4.5 (Mead et al., 1986). Hemocyte count was performed in a Neubauer chamber under an optical microscope. The negative controls consisted of uninfected, infected with *E. coli* HB101 and inoculated with sterile PBS larvae. This procedure was performed in four larvae individually for each strain at each time, and the hemocytes concentration was calculated as the mean of the tested larvae.

### Data and Statistical Analyses

To analyze virulence in *G. mellonella* model, survival curves were made using the Kaplan–Meier method. The Log-Rank test was performed to observe if there was a significant difference in survival curves and Student’s *t*-test was performed to analyze the survival percentage after 96 h, the difference in the production of hydrolytic enzymes, siderophores, and biofilm between the typical and atypical strains. The Spearman’s correlation and the chi-square tests were used to investigate if there was a correlation between the amount or kind of virulence genes and the high virulence *in vivo* after 96 h of infection, respectively. All analyses were performed using Graphpad Prism 6 and the degree of statistical significance considered was 95% (*p* < 0.05). The hierarchical clustering of the isolates according to the presence or absence of virulence genes was made with the program Excel Solver, with Matching coefficients and Group Average Linkage method.

## Results

### Genotypic and Phenotypic Characterization of EAEC Strains

#### Detection of Virulence Genes

Through PCR assay, typical strains had eight to 13 virulence genes (mean = 9.7 genes/strain), while atypical ones had zero to eight (mean = 2.6 genes/strain) (*p* < 0.0001). *Sen* and *sat* genes were not observed in any strain, while the *aap* and *set1A* were present in all typical strains (Table 2).

**TABLE 2:** Genotypic and phenotypic characterization of EAEC strains (gray square for positivity) and % survival of *G. mellonella* model 96 h after infection.

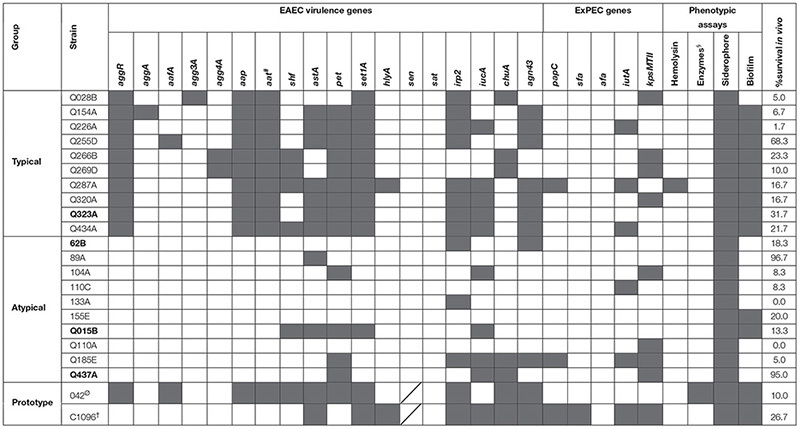

*The strains highlighted in bold were isolated from children with diarrhea. Crossed cells in the table indicate not determined. ^#^Lozer et al. (2013); ^§^ Enzymes – phospholipase, esterase, and protease. ^Ø^Czeczulin et al. (1999); Okeke et al. (2004); Boisen et al. (2008), and this study. ^†^Czeczulin et al. (1999) and this study.*

Considering ExPEC genes, of the 20 EAEC clinical strains analyzed, only two (one typical strain – Q287A – and one atypical strain – Q185E) had at least two of the five genes searched and were classified as possible ExPEC (Table 2). Of the prototype strains, only the atypical strain C1096 was classified as a possible ExPEC, since it presented four of those virulence markers (Johnson et al., 2003).

#### Siderophore and Hydrolytic Enzymes

No strains showed phospholipase, protease, and esterase activity under the employed conditions. The only strain containing the *hlyA* gene (strain Q287A – typical EAEC) produced hemolysin (Table 2), but in such a reduced amount that it did not allow quantification (data not shown). Regarding the prototype strains, only the strain 042 produced protease, being classified as a weak producer (*D*_colony_/*D*_halo_ = 0.76). In contrast, the mutant strain 042 did not show any enzymatic activity.

**FIGURE 1:**
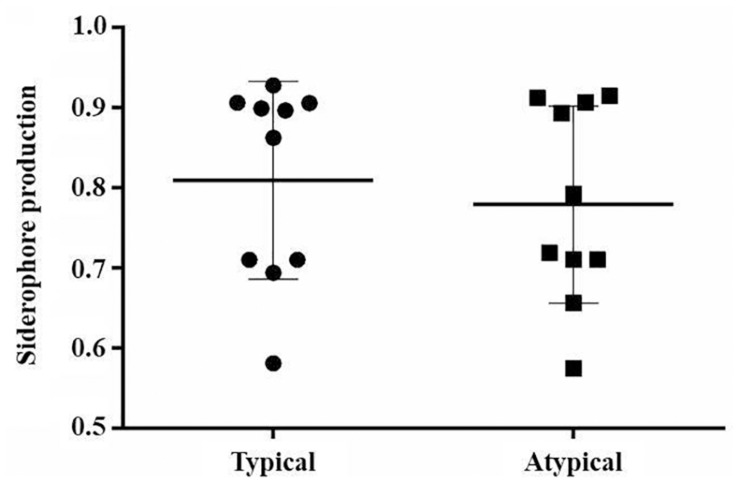
Siderophore production by EAEC strains, comparing typical and atypical groups (*p* > 0.05).

All analyzed strains produced siderophores. Sixteen strains were classified as “weak producers” and four were classified as “good producers” (*D*_colony_/*D*_halo_ < 0.7), two of them being typical EAEC and two being atypical. There was no significant difference in the production of siderophore between typical and atypical EAEC strains (*p* > 0.05) (Figure 1). All prototype strains were classified as “weak producers.”

#### Biofilm Production

**FIGURE 2:**
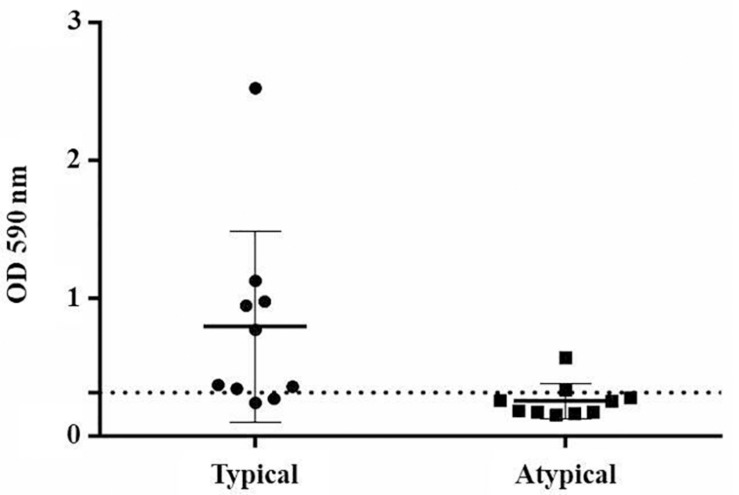
Biofilm production by EAEC strains, comparing typical and atypical groups (*p* < 0.05). The dotted line indicates the cut-off point to classify biofilm producers.

Eight strains of typical EAEC were able to produce biofilm, whereas only two strains of atypical EAEC could do the same (*p* < 0.05) (Figure 2).

Both typical and atypical prototype strains were classified as biofilm producers, although strain 042 produced biofilm with a density 6.7 times greater than the C1096. In contrast, the mutant strain 042 lost the ability to form a biofilm.

### *In vivo* Assays in *G. mellonella* Model and Relation With Genotypic and Phenotypic Aspects

#### *G. mellonella* Killing Assay

**FIGURE 3:**
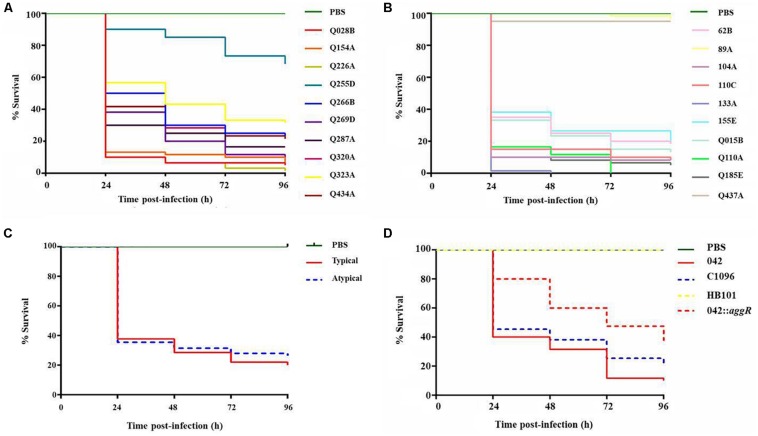
Survival curve graphs of the *G. mellonella* inoculated with clinical and prototype strains. **(A)** 10 typical EAEC strains analyzed at the concentration of 10^5^ CFU/larva. **(B)** 10 atypical EAEC strains analyzed at the concentration of 10^5^ CFU/larva. **(C)** Survival curve by groups of strains (typical and atypical) (*p* = 0.04). **(D)** Prototype strains EAEC 042 (typical), C1096 (atypical), non-pathogenic *E. coli* strain HB101, and EAEC 042 *aggR* mutant at the concentration of 10^5^ CFU/larva.

The strains of typical and atypical EAEC caused mortality of larvae in a classical inoculum-dependent manner, since the larvae showed low mortality with lower inoculum of 10^4^ CFU/larva (between 0 and 60%) and 100% mortality with higher inoculum of 10^6^ and 10^7^ CFU/larva (data not shown). Therefore, the concentration 10^5^ CFU/larva was the one that showed the highest differentiation among the strains, being this chosen for the virulence analyses. In addition, it was observed that the *G. mellonella* model is capable of differentiating levels of pathogenicity among EAEC strains (Figures 3A,B). Comparing the total of 600 analyzed larvae in each typical and atypical group, a significant difference occurred between the survival curves (*p* = 0.044) (Figure 3C).

The prototype strains of typical (EAEC 042) and atypical (C1096) EAEC showed high mortality in the model, with no significant difference between them (*p* > 0.05); while the non-pathogenic *E. coli* strain HB101 was not able to kill the larvae at any of the concentrations tested (Figure 3D). A significant reduction in the mortality was observed for the larvae inoculated with the EAEC *aggR* mutant strain 042 compared with the wild-type strain 042 at the concentration of 10^5^ CFU/larva (*p* = 0.0001). While the larvae inoculated with the wild strain showed only 40% of survival in the first 24 h, those inoculated with the mutant strain showed 80%. The *aggR* mutant strain also showed lower virulence than the atypical prototype strain C1096 (*p* = 0.016) (Figure 3D).

**FIGURE 4:**
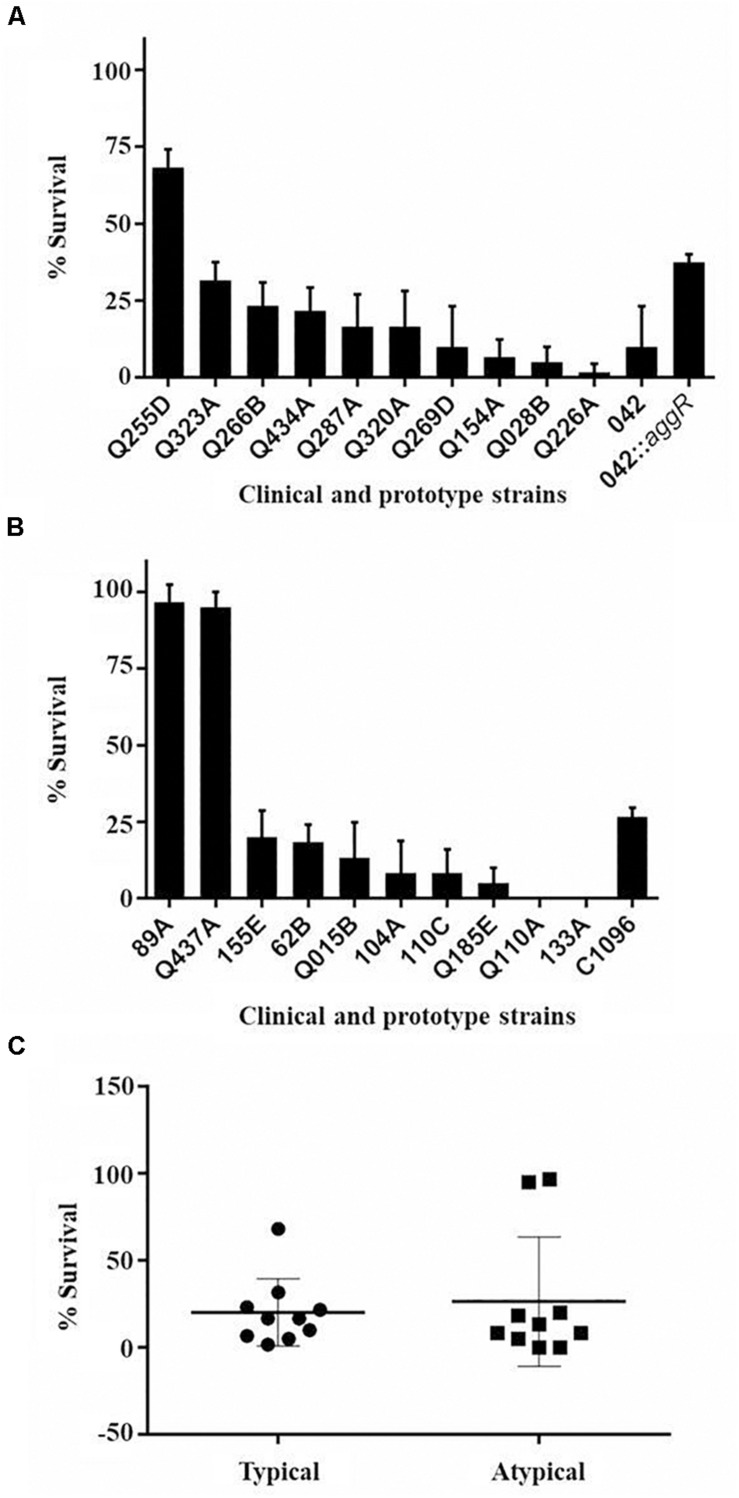
Percentage of survival of *G. mellonella* larvae after 96 h of infection. Percentage of survival of *G. mellonella* after 96 h of infection with typical **(A)** and atypical **(B)** EAEC strains at concentration 10^5^ CFU/larva. Data obtained from three independent experiments and expressed as mean ± SD. **(C)** Comparison of percentage of survival after 96 h of infection between the strains of typical and atypical EAEC (*p* > 0.05).

Among the clinical strains, atypical EAEC showed the highest and lowest virulence in the model, with 0% survival after 96 h of infection (Q110A and 133A strains, with only one virulence gene) or survival close to 100% at the end of the experiment (89A and Q437A, with one and four genes, respectively). Among the typical EAEC, only one strain (Q255D, with nine virulence genes) demonstrated a lower virulence, with survival greater than 60% at the end of 96 h (Table 2 and Figures 4A,B). There was no significant difference in the percentage of survival between typical and atypical strains after 96 h of infection (*p* > 0.05) (Figure 4C). Moreover, when analyzing the strains individually, it was observed that most of the atypical strains (8/10) showed high virulence in the model, similar to typical strains (9/10).

**FIGURE 5:**
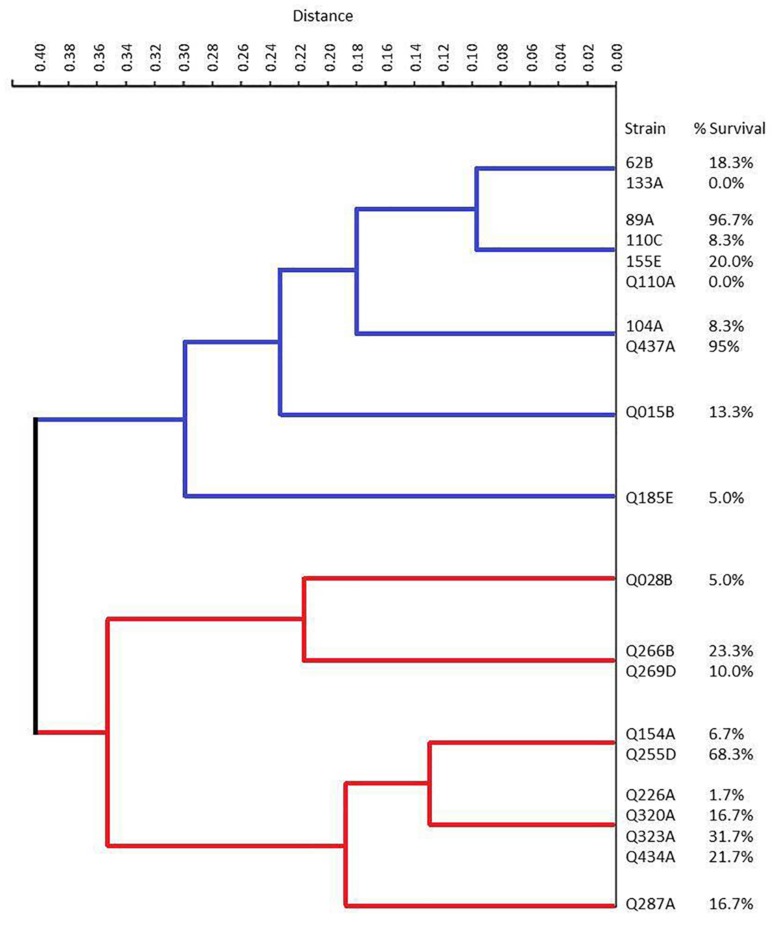
Dendrogram representation of the hierarchical clustering of the isolates according to the presence or absence of virulence gene with the corresponding EAEC strains and survival percentage. Red and blue lines represent the typical and atypical strains, respectively.

No statistical correlation was observed between the number of virulence genes and the percentage of survival after 96 h of infection (*r* = 0.12 and *p* > 0.05). In addition, no virulence gene or phenotypic aspect was individually related to high virulence *in vivo* (*p* > 0.05 for all genes and phenotypic aspects analyzed). Besides this, the hierarchical clustering based on the repertory of virulence genes showed a clear separation between typical and atypical strains, with strains grouped in the same subcluster presenting different virulence for the larvae (Figure 5).

#### Monitoring of Bacterial Growth *in vivo*

**FIGURE 6:**
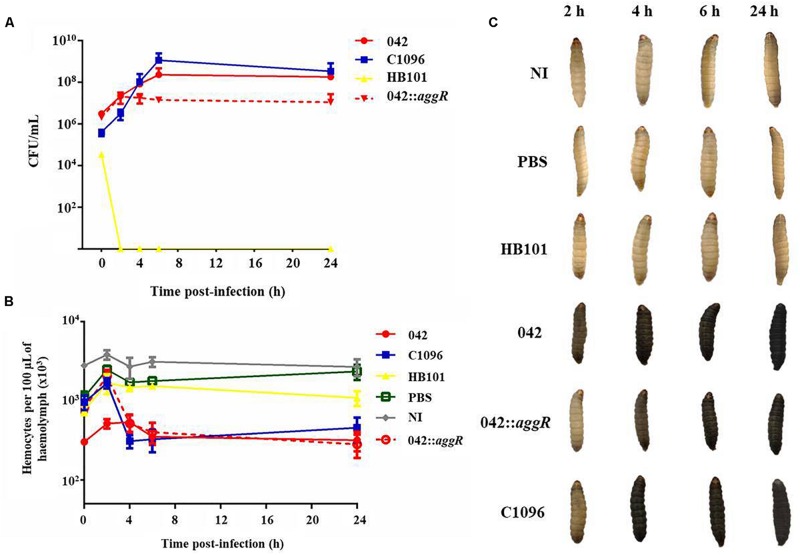
Monitoring of bacterial growth *in vivo*, hemocyte quantification and melanization of larvae during infection by EAEC prototype strains. **(A)** Quantification of the bacterial load in the hemolymph at 0, 2, 4, 6, and 24 h after infection with the prototype strains at 10^5^ CFU/larva. **(B)** Hemocyte quantification during infection by the prototype strains at 10^5^ CFU/larva using the uninoculated (NI) and inoculated with sterile PBS larvae as control. **(C)** Melanization of larvae during infection by EAEC prototype strains compared to larvae infected with non-pathogenic *E. coli* HB101, larvae inoculated with sterile PBS and uninoculated larvae (NI). Prototype strains: EAEC 042 (typical), EAEC 042 *aggR* mutant, EAEC C1096 (atypical), and non-pathogenic *E. coli* HB101.

The monitoring of bacterial growth, from zero up to 24 h after infection, showed that EAEC strains 042 (wild and *aggR* mutant) and C1096 growth within the larva, increasing its concentration in the first 6 h of infection followed by a stabilization of the bacterial load in 24 h. On the other hand, the non-pathogenic strain (HB101) was eliminated from the hemolymph within 2 h after infection. In addition, the *aggR* mutant strain showed a lower concentration in the hemolymph after 4 h of infection, when compared to the wild-type strain (Figure 6A).

#### Hemocyte Quantification During Infection

In the first 2 h after infection, there was an increase in the quantity of circulating hemocytes after all inoculations. After 4 h of infection, the concentration decreased for strain C1096 and, for strain 042, after 6 h; the *aggR* mutant behaved similarly to C1096 in all times analyzed (Figure 6B).

It was observed that only the larvae inoculated with both typical and atypical EAEC strains showed signs of melanization during infection, whereas the non-pathogenic *E. coli* strain HB101 did not induce this process (Figure 6C).

## Discussion

Typical EAEC are usually considered more virulent than atypical EAEC strains (Sarantuya et al., 2004; Morin et al., 2013). However, the outbreaks caused by the atypical ones (Čobeljić et al., 1996; Itoh et al., 1997), bring uncertainties regarding the difference in virulence among them. Therefore, to shed light on this issue the well-established *G. mellonella in vivo* model was used to compare virulence between typical and atypical EAEC clinical and prototype strains. We could show that atypical strains may be as virulent as typical strains, at least in the studied *in vivo* model, regardless of the analyzed genotypic or phenotypic aspects, but partially dependent of *aggR* for the prototype strain EAEC 042.

Initially, when establishing the optimal concentration of bacteria for the infection of *G. mellonella* larvae to the experiments, it was observed that larval death depends on the concentration of EAEC, as demonstrated in the first studies which analyzed clinical strains of typical EAEC in this model (Jønsson et al., 2016; Khalil et al., 2016). Although the killing capacity of the strain remains, our results do not perfectly match with those published previously by Jønsson et al. (2016). This was expected and is a current limitation of the *G. mellonella* model. The lack of universal genotypes of the larvae and the conditions adopted by each research group for feeding, reproduction, and maintenance of the animals can lead to quantitative differences in the results (Mowlds and Kavanagh, 2008; Olsen et al., 2011; Loh et al., 2013).

Although Sarantuya et al. (2004) proposed the *aggR* gene as a marker of truly virulent strains of EAEC, our experiments showed a similarity in virulence between typical and atypical strains in the *G. mellonella* model. However, the reduced mortality of the larvae infected with the *aggR* mutant EAEC strain 042 and the high virulence of atypical strains suggest that the virulence of EAEC seems to be related to the AggR regulon, but not exclusively. Corroborating with our results, a reduction in mortality was also observed for the *aggR* mutant EAEC strain 042 in the *Caenorhabditis elegans* model (França et al., 2013).

It is noteworthy that in the 042 strain, several other virulence factors are not under transcriptional control of AggR such as Pic (Protein involved in intestinal colonization), Pet (Plasmid encoded toxin), EAST (EAEC heat-stable enterotoxin 1), and irp2 (Yersiniabactin biosynthetic gene) (Morin et al., 2013). Pic, a serine protease secreted by EAEC, is able to cleave molecules of the complement system, among other phenotypes, indicating its role in systemic infections (Abreu et al., 2015), which is the case of our model. Since 042:*aggR* continues expressing Pic, the virulence presented by this mutant in *G. mellonella* could be due to Pic action on the complement system. In fact, *pic* deletion in one *E. coli* strain isolated from a case of sepsis led to attenuation of the strain in a murine model of sepsis (unpublished data).

Although most strains of atypical EAEC showed high virulence, the almost 100% survival after infection with two strains (89A, Q437A) suggests that their virulence genes (*astA*, *pet*, *iucA*, and *chuA*) were not responsible for this characteristic in this model, or, if any of them contributed to it, it might not have been expressed. Actually, the virulence in the *G. mellonella* model could not be attributed to any of the genes described in the EAEC strains other than *aggR* gene, as also observed by França et al. (2013) with *C. elegans* model.

The non-pathogenic *E. coli* strain HB101 was not able to kill the larvae at any of the concentrations tested, as has been demonstrated for other non-pathogenic strains of *E. coli* in *G. mellonella* (Leuko and Raivio, 2012; Pereira et al., 2015; Jønsson et al., 2016). Indeed, the HB101 strain was totally eliminated from the hemolymph by the immune system after 2 h of infection in opposition to the prototype strains of typical or atypical EAEC that lead to death of the larvae. In this case, these strains multiply in the larva, and mortality is possibly caused by the increase in the amount of bacteria inside it, as also previously demonstrated in studies with *Streptococcus pneumoniae*, *Actinobacillus pleuropneumoniae*, and EAEC 042 (Evans and Rozen, 2012; Pereira et al., 2015; Jønsson et al., 2016). Interestingly, the survival rate of larvae inoculated with commensal *E. coli* strains (10^5^ CFU/larva) was less than that of the HB101 strain but higher than that of the typical and atypical EAEC strains (data not shown). The adaptation and survival of commensal strains in human hosts probably gives greater virulence to larvae than the HB101 strain.

Infection by EAEC is accompanied by humoral immune response that leads to melanization of the *G. mellonella* larvae. After phagocytosis of the pathogen by hemocytes, melanin is deposited around microbes and is thought to facilitate bacterial killing (Browne et al., 2013). This response is observed after infecting *G. mellonella* with both typical and atypical EAEC, in which melanization began within 2 h after infection in the dorsal region of the larva, which contains the heart, and intensifies with the course of time. The larval dorsal vessel is an organ known to show a more intense immune response, due to the large amount of sessile hemocytes that are present on its surface and also of circulating hemocytes that are recruited together with the pathogen to the periphery of the dorsal vessel during infection (Pereira et al., 2015; Hillyer, 2018).

The cellular immune responses of *G. mellonella* were also investigated through hemocyte count and did not demonstrate discrepancy between typical and atypical EAEC prototype strains. There was an increase in the amount of circulating hemocytes, followed by a reduction. It may occur, on one hand, due to the cytotoxic activity of the bacteria on the cells, as demonstrated after infection of *G. mellonella* by *Pseudomonas aeruginosa* (Mizerska-dudka and Andrejko, 2014), and, on the other hand, due to their recruitment to the heart surface by the sessile hemocytes to aid the elimination of the pathogen (Pereira et al., 2015).

We also observed whether the *G. mellonella* model would be able to sort out strains at different levels of pathogenicity. However, although a large repertoire of virulence genes beyond phenotypic features was analyzed, no relation was observed. While the strain (Q255D) with nine of the studied genes had more than 60% survival, strains (Q110A and 133A) with one of the investigated genes including ExPEC genes, led to 100% mortality. Thus, factors currently unknown may be involved in the *in vivo* virulence of both typical and atypical EAEC.

Jønsson et al. (2016) suggested that AAF adhesin was related to the high virulence of EAEC in the *G. mellonella* model, by comparison with the Afa/Dr adhesin (Dr binding adhesins) which correlated with the pathogenicity of ExPEC (Ciesielczuk et al., 2015), although they did not perform any virulence assay of strains with and without AAF. Considering that, among the five the strains tested in our study that had some of the AAFs, four of them had a high virulence to the larvae (below 40% survival), and that most strains with high virulence (13/17) did not present the AAF genes, we suggest that additional factors could also be related to virulence beyond these genes, if in fact they participate in it. Therefore, the lack of relation between the virulence EAEC genes and virulence in the model leads us to suggest that unknown virulence factors may contribute to larval mortality.

In contrast, a study that analyzed the virulence of ExPEC in *G. mellonella* found a correlation between the number of virulence genes and the mortality of the larvae (Williamson et al., 2014). Our two EAEC strains with genes of ExPEC showed high virulence, as well as those without these genes.

In relation to the phenotypic characteristics of virulence, there was also no significant difference between the typical and atypical EAEC groups regarding the production of siderophores. Despite the importance of the enzymes in the pathogenesis of several microorganisms (Guignot et al., 2000; Ruiz-Perez and Nataro, 2014; Ramos et al., 2016), no significant production of these virulence factors by the analyzed strains was observed. Biofilm production, significantly associated with typical EAEC, was not related to *in vivo* virulence, since atypical strains that showed high virulence had no biofilm production. Interestingly, it was observed that the *aggR* mutant strain EAEC 042 did not show biofilm and protease production, such as the wild type strain 042, demonstrating the importance of this regulon for the expression of some virulence features. It has already been proven that the AggR regulon regulates several virulence genes, including genes involved in biofilm production (Morin et al., 2013).

The results obtained in this study demonstrate important aspects of the virulence of typical and atypical EAEC *in vivo*. It was observed that, contrary to what is suggested in the literature, atypical EAEC strains may be as virulent as typical ones, and their virulence is not due only to the AggR regulon and the genes regulated by it. Besides this, no relation was observed between phenotypic and genotypic characteristics of virulence and *in vivo* virulence in the *G. mellonella* model.

## Data Availability

The datasets for this manuscript are not publicly available because datasets are available on request. The raw data supporting the conclusions of this manuscript will be made available by the authors, without undue reservation, to any qualified researcher. Requests to access the datasets should be directed to LS, liliana.spano@ufes.br.

## Author Contributions

LS, RS, AS, and WE designed the study. CG, MP, and AG performed the experiments and analyzed the data. CG, LS, and WE wrote the manuscript. All co-authors reviewed and commented on the manuscript.

## Conflict of Interest Statement

The authors declare that the research was conducted in the absence of any commercial or financial relationships that could be construed as a potential conflict of interest.
